# Plant-Based Titanium Dioxide Nanoparticles Trigger Biochemical and Proteome Modifications in *Triticum aestivum* L. under Biotic Stress of *Puccinia striiformis*

**DOI:** 10.3390/molecules27134274

**Published:** 2022-07-02

**Authors:** Seema Hassan Satti, Naveed Iqbal Raja, Muhammad Ikram, Hesham F. Oraby, Zia-Ur-Rehman Mashwani, Azza H. Mohamed, Ajit Singh, Ahmad A. Omar

**Affiliations:** 1 Department of Botany, Pir Mehr Ali Shah (PMAS) Arid Agriculture University, Rawalpindi 46300, Pakistan; drnaveedraja@uaar.edu.pk (N.I.R.); ikramgondal464@gmail.com (M.I.); mashwani@uaar.edu.pk (Z.-U.-R.M.); 2 Deanship of Scientific Research, Umm Al-Qura University, Makkah 24381, Saudi Arabia; 3 Department of Crop Science, Faculty of Agriculture, Zagazig University, Zagazig 44519, Egypt; 4 Agricultural Chemistry Department, Faculty of Agriculture, Mansoura University, Mansoura 35516, Egypt; azza71@mans.edu.eg; 5 School of Biosciences, Faculty of Science and Engineering, University of Nottingham Malaysia, Semenyih 43500, Selangor, Malaysia; ajit.singh@nottingham.edu.my; 6 Biochemistry Department, Faculty of Agriculture, Zagazig University, Zagazig 44519, Egypt; 7 Citrus Research and Education Center (CREC), Institute of Food and Agricultural Sciences (UF/IFAS), University of Florida, Lake Alfred, FL 33850, USA

**Keywords:** bioinspired titanium dioxide nanoparticles, proteomic analysis, protein upregulation, biochemical profiling, *Puccinia striiformis*, antioxidants

## Abstract

In this study, we evaluated bioinspired titanium dioxide nanoparticles (TiO_2_ NPs) that elicited biochemical and proteome modifications in wheat plants under the biotic stress caused by *Puccinia striiformis f*. sp. *tritici* (*Pst*). Biosynthesis of TiO_2_ NPs was confirmed using UV–Vis spectrophotometry, energy dispersive X-ray spectroscopy (EDX), scanning electron microscopy (SEM), and Fourier transform infrared (FTIR) spectroscopy. We found that the nanoparticles with crystalline nature were smaller than 100 nm. The results of FTIR analysis showed the presence of potential functional groups exhibiting O-H, N-H, C-C, and Ti-O stretching. The TiO_2_ NPs of different concentrations (20, 40, 60, and 80 mg L^−1^) were exogenously applied to wheat plants under the biotic stress caused by *Pst*, which is responsible for yellow stripe rust disease. The results of the assessment of disease incidence and percent disease index displayed time- and dose-dependent responses. The 40 mg L^−1^ TiO_2_ NPs were the most effective in decreasing disease severity. The bioinspired TiO_2_ NPs were also evaluated for enzymatic (superoxide dismutase (SOD), peroxidase (POD), and catalase (CAT)), and nonenzymatic metabolites (total proline, phenolic, and flavonoid contents) in wheat plants under stripe rust stress. The 40 mg L^−1^ TiO_2_ NPs were effective in eliciting biochemical modifications to reduce biotic stress. We further evaluated the effects of TiO_2_ NPs through gel- and label-free liquid chromatography-mass spectrometry (LC-MS) proteome analysis. We performed proteome analysis of infected wheat leaves and leaves treated with 40 mg L^−1^ TiO_2_ NPs under stripe rust stress. The functional classification of the proteins showed downregulation of proteins related to protein and carbohydrate metabolism, as well as of photosynthesis in plants under biotic stress. An upregulation of stress-related proteins was observed, including the defense mechanisms and primary metabolic pathways in plants treated with 40 mg L^−1^ TiO_2_ NPs under stress. The experimental results showed the potential of applying biogenic TiO_2_ NPs to combat fungal diseases of wheat plants and provided insight into the protein expression of plants in response to biotic stress.

## 1. Introduction

Agriculture plays an important role in the commercial, economic, and social advancement in developing countries. Wheat is a staple and a valuable food crop that accounts for approximately 30% of the world’s cereal production; wheat is a main source of food. Wheat production should be increased 60% by 2050 to fulfill the food demands of the growing population. Wheat is a basic source of essential carbohydrates and proteins, and feeds millions of people worldwide. Unfortunately, wheat production is decreasing due to various fungal and bacterial diseases. Fungal diseases cause huge wheat losses by damaging its physiological, biochemical, and antioxidant defense systems, ultimately resulting in low food quality and quantity [1].

Stripe or yellow rust is caused by the fungus *Puccinia striiformis* f. sp. tritici (*Pst*). *Pst* is the most damaging wheat disease, affecting root growth, plant height, number of grains per spike, and, consequently, decreased dry matter, yield, and grain quality [2,3]. The average wheat yield loss caused by the yellow rust ranges from 25% to 80% worldwide [4].

The uses of fungicides and resistant varieties are effective against yellow rust. However, the prolonged use of fungicides has hazardous effects on crops and, ultimately, on human health and ecosystems. Furthermore, pathogens, especially *Pst*, dynamically generate new virulent genes (pathogen variants) in the pathogen population, leading to decreased host resistance. Plant growth, development, and reproduction are affected by biotic factors at various levels of disease severity [5,6].

The essential plant defense systems protect cells from pathogen invasions. Environmental adversities induce the aggregation of reactive oxygen species (ROS) in the cells, resulting in severe oxidative damage, which inhibits plant growth and, therefore, yields. ROS also play a role in programmed cell death and act as signal inducers in various cellular and physiological pathways [7,8]. Moreover, through several other routes, the production of ROS induces the activation of plant-defense-related compounds, including enzymatic (SOD, POD, and CAT) and nonenzymatic (flavonoids, phenolic, and proline) antioxidants for ROS scavenging [9,10]. However, if the imbalance between ROS synthesis and scavenging exceeds the abilities of the defense mechanism, oxidative stress increases, ultimately contributing to yield losses. Furthermore, elevated ROS levels result in irreversible damage to the physiological capacity of the cell and eventually lead to cell death [8].

Nanotechnology involves the use of nanomaterials in agriculture to develop methods that limit the use of harmful agrochemicals and help boost the yield of different crops [11,12]. Among the metal oxide nanomaterials, titanium dioxide nanoparticles (TiO_2_ NPs) have been extensively used as a clean photocatalyst due to their plasmonic nature, high chemical stability, and nontoxicity [13,14,15]. Due to their photocatalytic properties, TiO_2_ NPs exhibit significant antifungal and antibacterial activities, which can be employed in the agriculture sector to protect plants from pathogenic infections [16].

TiO_2_ NPs are considered antimicrobial agents because of their photocatalytic nature, low toxicity, cost-effectiveness, and chemical stability. Furthermore, TiO_2_ NPs help to inactivate various microorganisms due to their strong oxidizing power producing free radicals, such as superoxide anion radicals, thereby reducing the growth of microorganisms. Moreover, TiO_2_ NPs possess excellent larvicidal, antimicrobial, wound healing, antioxidant, and anticancer abilities, and play a notable role in the medical field [17]. Recently TiO_2_ NPs showed excellent antimicrobial activity against various bacterial strains, i.e., *Bacillus subtilis*, *Klebsiella pneumonia*, and *Staphylococcus*; and fungal strains, i.e., *Aspergillus flavus*, *Rhizopus oryzae*, *Sclerotium rolfsii*, and *Aspergillus niger* [17].

Generally, nanoparticles elicit plant defense mechanisms and provide an alternative approach to combating proliferating fungal pathogens. Nowadays, understanding the plant defense mechanism against fungal pathogens has become important. Various studies have indicated that the massive reallocation of energy and its efficacious supply are imperative for plant defense processes in response to pathogen invasion [7,8,18]. Therefore, energy-related metabolic pathways and plant defense mechanisms exhibit coordination in response to biotic stresses [19]. Proteins are directly associated with cell physiological processes. Thus, proteomic analyses contribute to comparing the protein abundance between resistant and susceptible as well as control and stressed crops. The induction of stress alters gene expression, thereby regulating physiological and metabolic mechanisms, causing changes in intracellular protein expression [20,21,22]. Therefore, knowledge of protein functions and their alterations in response to biotic stress is essential in understanding the molecular processes underlying stress resistance.

In the present study, we used biogenic TiO_2_ NPs to control the proliferation of the pathogenic fungus *Pst*. The response of *Pst*-stressed wheat plants was measured in terms of biochemical parameters. Gel- and label-free proteome analysis was performed to compare protein expression among the pathogen-free control, pathogen-affected, and TiO_2_-NPs-treated wheat plants under *Pst* infection. The bioinformatics were analyzed to determine the functional categorization of proteins.

## 2. Results and Discussion

### 2.1. Biogenesis and Optical Characterization of TiO_2_ NPs

**Figure 1:**
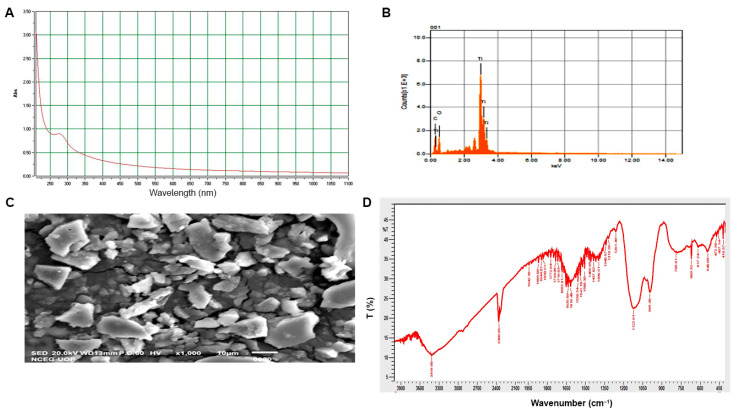
Physical and optical characterization of biogenic TiO_2_ NPs: (**A**) UV–visible spectrum; (**B**) elemental composition analysis; (**C**) SEM image; (**D**) Fourier transformed infrared (FTIR) spectroscopy.

The biogenic synthesis of TiO_2_ NPs is superior to other common physical and chemical methods of NPs synthesis due to the one-step reaction, ecofriendly nature of the reactants, biocompatible product, and cost-effectiveness. Plant secondary metabolites play a role in reducing and stabilizing bulk materials in redox reactions [23]. In this study, we used *Moringa oleifera* leaf aqueous extract to reduce the Ti(OH)_2_ salt into TiO_2_ NPs. Initially, the synthesis of TiO_2_ NPs was observed by the reaction mixture changing color from milky white to pinkish brown, which is a characteristic of TiO_2_ NPs synthesis. The synthesis of TiO_2_ NPs was confirmed by observing the light absorbance of the colloidal suspension in terms of surface plasmon resonance (SPR)-band using UV–visible spectrophotometry. A characteristic SPR band was observed, and the highest peak ranged between wavelengths of 300 and 450 nm (Figure 1A) [12]. Our results are in line with the findings of Mustafa et al. [1], who reported that characterization peaks range between 250 and 300 nm. These results are in agreement with those of Satti et al. [12].

The elemental composition of TiO_2_ NPs was determined by EDX analysis. Characteristic titanium peaks were observed in the range of 4–5 keV (Figure 1B). Our observations of other elements were due to the number of organic compounds found in the leaf extract used for reducing the TiO_2_ salt.

### 2.2. Physical and Biochemical Characterization of TiO_2_ NPs

TiO_2_ NPs were structurally observed using the SEM. The images of the biogenic TiO_2_ NPs showed that the nanoparticles were irregular in shape. However, some of the nanoparticles were cylindrical or rectangular (Figure 1C). Most of the nanoparticles were also observed in the form of small clusters. These results are in accordance with those of other studies that reported green, irregular and spherical, synthesized TiO_2_ NPs with sizes ranging between 25 and 100 nm [24].

FTIR was performed to identify the various biomolecules used for the synthesis, capping, and stabilizing of the TiO_2_ NPs (Figure 1D). The results of FTIR analysis depicted some peaks at 1437 and 1616 cm^−1^, indicating the presence of a carbonyl group (C=O) and C=C aromatic ring, respectively. These functional groups are involved in the reduction of salts into titanium dioxide nanoparticles. The peaks around 3419, 1465, 1319, and 1100 cm^−1^ indicated OH groups, a carbonyl group, stretching of the C=C aromatic ring, and stretching vibrations of the C–OH group, respectively. Further peaks around 1400 cm^−1^ were generally attributed to the bending vibration of H–OH groups in TiO_2_ [25].

### 2.3. Assessment of Disease Severity Caused by the Pst

**Figure 2:**
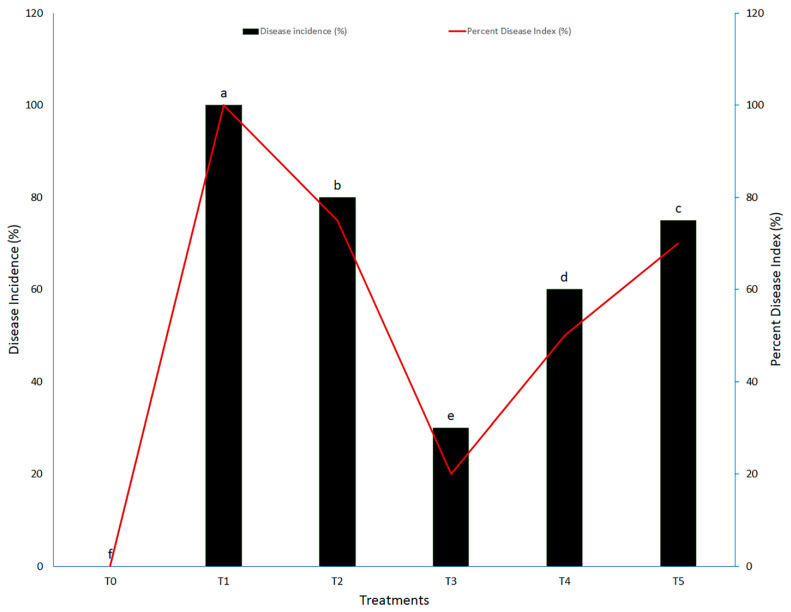
Disease incidence and percent disease index of wheat plants infected with *Puccinia striiformis*. Treatments: T_0_: control (healthy wheat plants), T_1_: plants infected with pathogen (*Puccinia striiformis)* only, T_2_: plants infected with pathogen + 20 mg L^−1^ TiO_2_ NPs, T_3_: plants infected with pathogen + 40 mg L^−1^ TiO_2_ NPs, T_4_: plants infected with pathogen + 60 mg L^−1^ TiO_2_ NPs; T_5_: plants infected with pathogen + 80 mg L^−1^ TiO_2_ NPs. Different small letters indicate significant differences in results (*p <* 0.05).

The disease incidence and percent disease index were measured against the *Pst* using various concentrations of biogenic TiO_2_ NPs in intervals of different days. Biogenic TiO_2_ NPs had different effects on the occurrence of yellow rust disease in wheat plants, which were dependent on the nanoparticle concentration and time elapsed since their first application. The first record was collected after the 10th day of inoculation, and the same was repeated every 10 days until harvest. It was experimentally evident that none of the applied TiO_2_ NPs concentrations fully inhibited the proliferation of yellow rust fungus (*Pst*). However, each concentration differently affected disease severity. The recorded disease incidence of yellow rust continually declined over time in all treatments with biogenic TiO_2_ NPs (Figure 2). 

The maximum values of disease incidence and percent disease index were found in the wheat plants that were suffering from fungal infection but not treated with biogenic TiO_2_ NPs (Figure 2). However, the biogenic applications of TiO_2_ NPs inhibited the further growth and proliferation of the fungal pathogen, which resulted in decreased disease incidence and percent disease index when treated with 40 mg L^−1^ of TiO_2_ NPs. The values of the percent disease index and disease incidences decreased by up to 74% and 65%, respectively, in wheat plants infected with yellow rust disease exposed to 40 mg L^−1^ of TiO_2_ NPs (Figure 2). According to our results, we observed that TiO_2_ NPs had differential effects and showed dose-dependent responses on the disease severity and disease incidence in wheat plants. The 40 mg L^−1^ TiO_2_ NP treatment was the most significant in creating resistance against stripe rust disease in wheat plants. The incidence of stripe rust progressively decreased when 20 and 40 mg L^−1^ of TiO_2_ NPs were applied. Furthermore, the incidence increased with increasing application concentration of TiO_2_ NPs, which indicated the toxic nature of those concentrations on the immune system of the plants. Our findings are in line with those of Satti et al. [12], who reported that TiO_2_ NPs showed time- and dose-dependent responses in wheat plants under biotic stress. According to their findings, TiO_2_ NPs at concentrations of 20 and 40 mg L^−1^ reduced biotic stress in wheat plants; enhanced the agronomic-, biochemical-, and defense-related antioxidant enzymes; and improved plant health. However, they also reported that TiO_2_ NPs may be toxic, as the concentration of nanoparticles greatly affected the wheat plants. Unfortunately, limited data are available on the cytotoxicity of TiO_2_ NPs, which should further be explored. TiO_2_ NPs possess antimicrobial and photocatalytic activities, and showed an effective ability to control and suppress various plant infections caused by different pathogens [26,27,28]. For example, Haghighi et al. [29] reported that TiO_2_ NPs exhibited an antifungal effect against fluconazole-resistant strains of *Candida albicans*.

Due to their small size and physicochemical attributes, TiO_2_ NPs play a significant role in suppressing different infections caused by pathogens [30]. This small size enables the particles to enter the plasma membrane, destabilize the fungal hyphae, and thus affect cell homeostasis. This mechanism also disturbs the membrane integrity, resulting in cytoplasm leakage, which inhibits physiological processes and ultimately causes fungal cell death. These events also lead to the entry of TiO_2_ NPs to the fungal cell nucleus, causing oxidation, which leads to fragmentation of fungal DNA and eventually cell death. Additionally, TiO_2_ NPs affect other physiological pathways, contributing to their antimicrobial mechanism. The biochemical pathways influenced by TiO_2_ NPs include enzyme action inhibition, disorganization of the electron transport chain, disruption of ATP synthase activity, mitochondrial enzyme oxidation, inhibited cellular signaling pathways, and cell surface receptor blockage [31].

### 2.4. Assessment of Plant Biochemical Attributes against Pst Stress in Response to TiO_2_ NPs 

**Figure 3:**
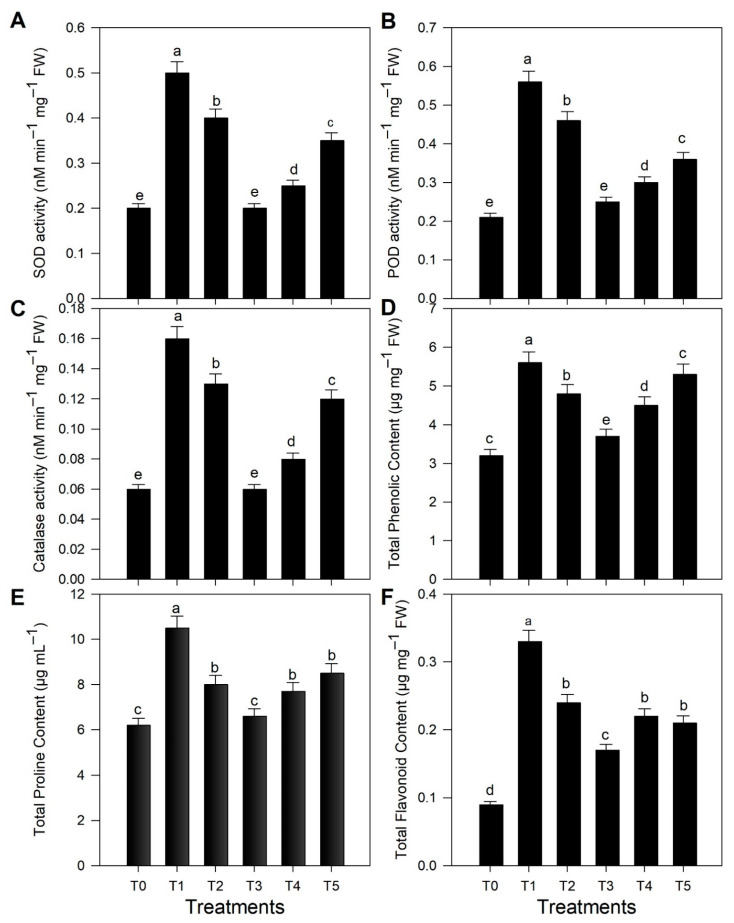
Effect of exogenous application of TiO_2_ NPs on the biochemical profile of wheat plants infected with *Puccinia striiformis:* (**A**) SOD, (**B**) POD, and (**C**) catalase activities; (**D**) total phenol, (**E**) total proline, and (**F**) total flavonoid contents. Treatments: T0: control (healthy wheat plants), T_1_: plants infected with pathogen (*Puccinia striiformis)* only, T_2_: plants infected with pathogen + 20 mg L^−1^ TiO_2_ NPs, T_3_: plants infected with pathogen + 40 mg L^−1^ TiO_2_ NPs, T_4_: plants infected with pathogen + 60 mg L^−1^ TiO_2_ NPs, and T5: plants infected with pathogen + 80 mg L^−1^ TiO_2_ NPs. Different small letters indicate significant differences in results (*p <* 0.05).

Crop yield and quality depend on the ability of plants to cope with various types of biotic and abiotic environmental stresses. Reactive oxygen species (ROS) start to accumulate due to these environmental stresses in plant cells; as a result, plants suffer from severe oxidative damage, which affects growth, productivity, and yield [11,12,32]. Plants possess various defense mechanisms that are activated during stress. Different enzymatic (superoxide dismutase, catalase, and peroxidase) and nonenzymatic (flavonoids compounds, proline, and phenolic) antioxidant pathways result in the activation of several defense mechanisms against oxidative damage [7,8,33]. In this study, both enzymatic (SOD, POD, and CAT) and nonenzymatic (proline content, total flavonoid content, and total phenolic content) antioxidants were evaluated to determine the antifungal activity of the biogenic TiO_2_ NPs in response to stripe rust disease in wheat. Increases in the production rates of SOD, POD, and CAT were observed in the fungal-infected wheat plants (Figure 3A–C). The activities of SOD (0.5 nM min^−1^ mg^−1^ FW) and POD (0.56 nM min^−1^ mg^−1^ FW) in wheat plants were affected by stripe rust stress. Both SOD and POD exhibited dose-dependent activity, and showed a linear relationship with CAT activity. A significant CAT activity was found (0.16 nM min^−1^ mg^−1^ FW) in stripe-rust-infected wheat plants without treatment with TiO_2_ NPs. TiO_2_ NPs application reduced endogenous enzyme production. The maximum reduction in enzyme concentration was observed in the infected plants in response to applying 40 mg L^−1^ of TiO_2_ NPs (Figure 3A–C). Okupnik and Pflugmacher [34] observed that TiO_2_ NPs stimulated the production of glutathione reductase and catalase in water thyme plants. The application of nanoparticles, especially TiO_2_ NPs, may help reduce biotic stress, leading to the reduction in the production of these endogenous enzymes [8]. However, the efficacy of TiO_2_ NPs showed a dose-dependent tendency. Various studies reported decreases in the growth-related and physiological attributes of various plants in response to higher concentrations of TiO_2_ NPs [12]. TiO_2_ NPs were also reported to enhanced superoxide dismutase, peroxidase, and catalase activities at higher concentrations, whereas plant cell membranes showed serious damage upon exposure to TiO_2_ NPs concentrations higher than 500 mg L^−1^ [35].

Our experimental evidence showed a substantial production of proline in wheat plants infected with yellow rust disease. However, the foliar application of TiO_2_ NPs improved the proline content of the diseased plants. The proline content was highly evident (10.5 µg mL^−1^) in infected wheat plants, and was reduced in the plants exposed to 40 mg L^−1^ of TiO_2_ NPs (Figure 3E).

The nonenzymatic phenolic and flavonoid contents were also investigated in wheat plants affected by stripe rust stress upon the exogenous application of biogenic TiO_2_ NPs (Figure 3E,F). The TPC increased in wheat plants under biotic stress (5.6 µg mg^−1^ FW) (Figure 3D). However, the application of nanoparticles decreased the phenolic content levels. A maximum decrease was recorded in the wheat plants treated with a 40 mg L^−1^ of TiO_2_ NPs foliar spray. A similar trend was observed for the total flavonoid content, which also increased in plants under stress, and significantly reduced when biogenic TiO_2_ NPs were applied. A marked reduction in the TFC was recorded in the plants sprayed with 40 mg L^−1^ of TiO_2_ NPs (Figure 3F). Few scientific data are available on the role of TiO_2_ NPs in the modification of nonenzymatic attributes, i.e., TPC and TFC, in wheat plants. A study reported that TPC and TFC increased in rice plants under biotic stress. However, plant-based silver nanoparticles decreased nonenzymatic activities in rice suffering from biotic stress. Reactive oxygen species are produced in plants under biotic stresses. To cope with this situation, plants enhance their enzymatic and nonenzymatic activities. The foliar applications of nanomaterial enhanced the antioxidant defense system of plants and scavenged the level of reactive oxygen species by lowering the TPC and TFC, which indicated stress alleviation in wheat plants. TPC and TFC play a significant role in the protection of plants under biotic and abiotic stresses by lowering the levels of reactive oxygen species. Furthermore, they are also responsible for maintaining the homeostasis and organelle structure in plants [12,36].

These results helped to establish a direct relationship between the applications of TiO_2_ NPs and biotic stress caused by *Pst*. Wheat plants under biotic stress exhibited marked increases in the SOD, CAT, POD, and proline contents, and TFC and TPC in the infected plants. However, the exposure to TiO_2_ NPs led to biochemical, osmotic, and enzymatic adjustments, which were necessary to cope with the biotic stress and inhibit the spread of *Pst*.

### 2.5. Principal Component Analysis (PCA) and Two-Way Hierarchical Cluster Analysis (HCA)

**Figure 4:**
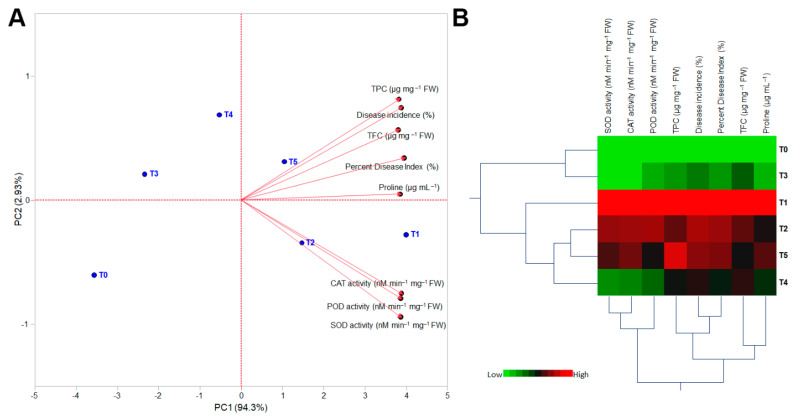
Principal component analysis (PCA) and two-way hierarchical cluster analysis (HCA) of wheat plants inoculated with *Puccinia striiformis* and treated with different concentrations of TiO_2_ NPs in association with biochemical parameters, disease incidence, and percent disease index. (**A**) PCA-associated scatter plots and loading plots (Biplot), and (**B**) two-way HCA. The heat map visualizes variations. Rows correspond to the TiO_2_ NPs treatments, whereas columns correspond to different measured parameters. Treatments: T_0_: control (healthy wheat plants), T_1_: plants infected with pathogen (*Puccinia striiformis)* only, T_2_: plants infected with pathogen + 20 mg L^−1^ TiO_2_ NPs, T_3_: plants infected with pathogen + 40 mg L^−1^ TiO_2_ NPs, T_4_: plants infected with pathogen + 60 mg L^−1^ TiO_2_ NPs, and T_5_: plants infected with pathogen + 80 mg L^−1^ TiO_2_ NPs. The heat map is colored according to numerical values, where low values are green and high values are red.

Both principal component analysis (PCA) and hierarchical cluster analysis (HCA) were conducted to better understand the results (Figure 4). On the PCA-associated scatter plot, we observed a strong separation between the TiO_2_ NPs treatments in terms of PC1 (about 94.3%) and PC2 (around 2.93%) (Figure 4A). Furthermore, the PCA-associated loading plot indicated that nonenzymatic antioxidants (TPC, TFC, and proline), disease incidence (%), and percent disease index (%)more positively correlated with T5 (plants infected with pathogen + 80 mg L^−1^ TiO_2_ NPs), whereas enzymatic antioxidants (SOD, CAT, and POD) were associated with T1 (plants infected with pathogen (*Puccinia striiformis*) only) and T2 (plants infected with pathogen + 20 mg L^−1^ TiO_2_ NPs) (Figure 4A).

The heatmap of the HCA, which demonstrated the differences among treatments of TiO_2_ NPs, agreed with the PCA results (Figure 4B). Briefly, the dendrogram of HCA among the treatments showed that they were separated into three distinct clusters. The first cluster included T0 (control) and T3 (plants infected with pathogen + 40 mg L^−1^ TiO_2_ NPs); the second cluster included only T1; and the third cluster consisted of T2, T3, and T4. Additionally, the dendrograms of the HCA-associated measurements showed that all parameters clearly fit into three distinct clusters. The first cluster included the enzymatic antioxidants (SOD, CAT, and POD); disease incidence (%), percent disease index (%), and total phenolic content were in the second cluster. The third cluster included the nonenzymatic antioxidants (total flavonoid content and total proline content) (Figure 4B).

### 2.6. Proteome Analysis of Wheat Plants in Response to TiO_2_ NPs under Biotic Stress Caused by Pst

The best concentrations of *Moringa oleifera* Lam mediated biosynthesized TiO_2_ NPs were selected for the proteome analysis from the data derived from the glasshouse experiment. The data revealed that 40 mg L^−1^ of TiO_2_ NPs was the most effective concentration against the biotic stress caused by *Pst* in wheat plants, as it showed the maximum disease resistance against yellow stripe rust. We performed proteome analysis to determine the differential proteins in wheat under biotic stress compared with those in plants receiving the 40 mg L^−1^ of TiO_2_ NPs treatment under biotic stress, and to compare these two treatments with the control. The flag leaves for each treatment were harvested for the proteome analysis using the gel- and label-free nano-LC-MS/MS technique. 

### 2.7. Proteome Analysis of Wheat Plant under Pst Stress

**Figure 5:**
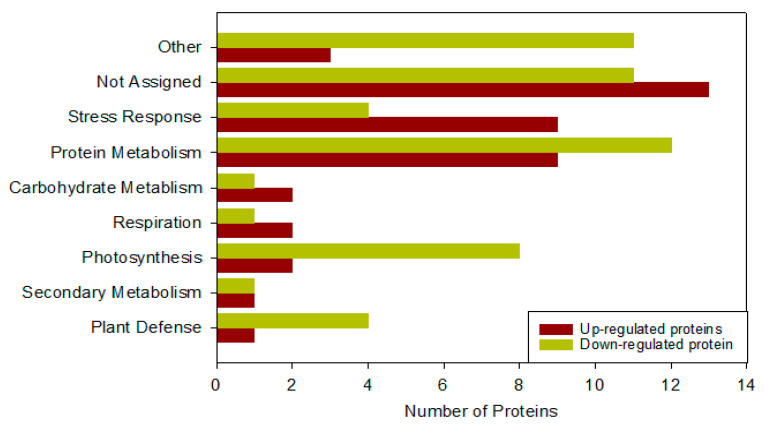
Bar diagram of the functional categorization of up- and downregulated proteins under *Puccinia striiformis* stress in comparison with the control. X-axis shows the observed number of up- and downregulated proteins. Others include metabolism proteins, stress, plant defense, photosynthesis, respiration, lipid metabolism, carbohydrate metabolism, development, transport, signaling, cell, secondary metabolism, miscellaneous, not assigned, and others.

The proteome analysis of flag leaves of plants infected with *Pst* was performed without applying nanoparticles to evaluate the significant changes in proteins and to compare the results with those of the control plants. A total of 95 proteins were recognized. We used MapMan Bin codes software (https://mapman.gabipd.org/home, accessed on 1 May 2022) to categorize the functions of the identified proteins (Figure 5). Those were further divided into 42 upregulated and 53 downregulated proteins (Tables S1 and S2). The functional groups into which the proteins were divided included proteins associated with cell division, chromatin, defense and stress, transport of ions, protein metabolism pathways, growth and development, lipid metabolism, carbohydrate metabolism, transcriptional modification, and a few others with known or unknown biophysiological functions. The results proteome analysis of wheat plants under biotic stress showed that the proteins associated with protein metabolism, photosynthesis, carbohydrate metabolism, and defense were downregulated. Conversely, the proteins related to stress, such as Zn/Cu superoxide dismutase, thylakoid L. ascorbate, ribulose bisphosphate carboxylase respiration, and transport, were upregulated (Figure 5).

### 2.8. Proteome Analysis of Wheat Plants under Pst Stress in Response to 40 mg L^−1^ of TiO_2_ NPs

**Figure 6:**
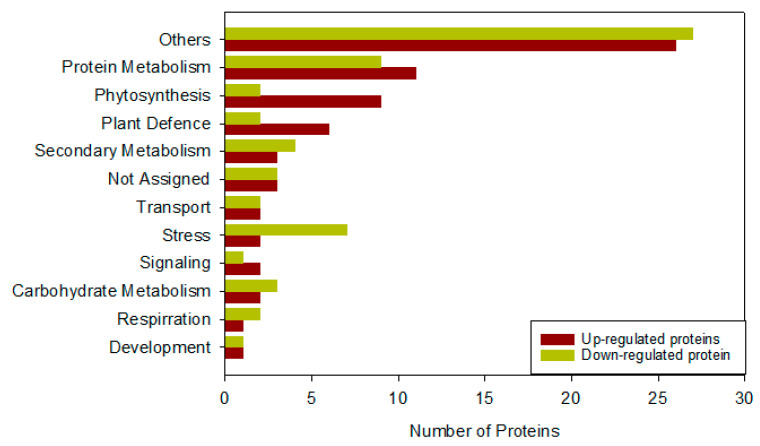
Functional categorization of up-and downregulated proteins in wheat plants sprayed with 40 mg/L of TiO_2_ NPs under *Puccinia striiformis* stress. X-axis shows the relative number of up- and downregulated proteins. Others include protein metabolism, stress, plant defense, photosynthesis, respiration, lipid metabolism, carbohydrate metabolism, development, transport, signaling, cell, secondary metabolism, miscellaneous, not assigned, and others.

We performed proteome analysis of the flag leaves of wheat plants under biotic stress caused by *Pst* in response to the application of 40 mg L^−1^ of TiO_2_ NPs to evaluate the changes in proteins and to compare the results with those of control. The analysis led to the documentation of a total of 121 proteins. We used MapMan codes software to categorize the functions of these identified proteins (Figure 6). They were divided into 63 upregulated and 58 downregulated proteins (Tables S3 and S4). The functional groups included proteins associated with cell division, chromatin, defense and stress, transport of ions, protein metabolism pathways, growth and development processes, lipid metabolism, carbohydrate metabolism, transcriptional modification, and a few others with known or unknown biophysiological functions. The results of the analysis also showed that the proteins associated with protein metabolic pathways, photosynthesis, carbohydrate metabolism, and defense (rubisco activase, peroxidase, catalase-1, and ascorbate peroxidase) were upregulated. Meanwhile, those related to stress (Zn/Cu superoxide dismutase, thylakoid L. ascorbate, and ribulose bisphosphate carboxylase), respiration, and transport were downregulated (Figure 6).

The results showed an extreme inhibitory impact of the biotic stress due to *Pst* on the morphological, physiological, and biochemical components of wheat plants compared with the control. This inhibitory impact adversely affected the quantity and quality of grains, rendering them unhygienic and harmful for human consumption. However, the foliar spraying of TiO_2_ NPs during various stages of wheat plants produced improvements in all physiological and biochemical attributes, resulting in the induction of resistance to stripe rust. The results revealed that the maximum positive effects were produced by the 40 mg L^−1^ TiO_2_ NPs sprayed on wheat plants affected by biotic stress. The 40 mg L^−1^ of TiO_2_ NPs were selected for the proteome analysis to understand the underlying mechanisms of the protein dynamics of biotic stress and after 40 mg L^−1^ TiO_2_ NPs treatment of wheat plants. The size and properties of nanoparticles determine their entry into plants. Nanoparticles can enter the cell through pits in the cell wall and effectively pass to the plasma membrane [37]. The size of the nanoparticle also determines their rate of entry, as large nanoparticles may face difficulties in entering or cannot enter the cell wall pores [38]. Nanoparticles can penetrate only through the stomata, flower stigma, or hydathodes. After entering the plant cell, nanoparticles act as metallic ions, react with the amino acids, and change protein activities.

The data regarding proteome analysis showed the upregulation of proteins associated with stress. We also observed an upregulation of proteins related to protein production and downregulation of proteins associated with photosynthesis and carbohydrate mechanisms. However, the plants treated with 40 mg L^−1^ of TiO_2_ NPs under biotic stress showed an upregulation of various defense-related proteins and upregulated photosynthesis-related proteins. The proteins related to protein metabolism and stress were downregulated upon treatment with 40 mg L^−1^ of TiO_2_ NPs. The promotion of photosynthesis could have been due to the enhanced activity of photosynthesis-related enzymes. The results from this study showed an upregulation of rubisco in response to TiO_2_ NP application against biotic stress. The enhanced activity of rubisco in spinach was also reported by Yang et al. [39] in response to TiO_2_ NPs applications. In various studies, TiO_2_ NPs had a promoting effect on the Hill reaction. Hence, they trigger the activities of chloroplast enzymes, resulting in increased photosynthetic activity. The entry of nanoparticles into chloroplasts causes an increased rate of electron transport, thereby enhancing the production of oxygen, and consequently increasing the quotient of oxidation-reduction reactions [39,40,41]. TiO_2_ NPs were also reported to possess photocatalytic properties, which enable them to enhance the light absorbance that enriches the assimilation of carbon dioxide [42].

Wheat plants show resistance against pathogen attack by implementing a variety of defense responses, including various processes such as the accumulation of ROS. Zhang et al. [41] described a similar mechanism that involved the accumulation of H_2_O_2_ in palisade mesophyll cells of barley plants attacked by powdery mildew. In this study, several defense-related proteins, such as putative glutathione, transferase, and glutathione, were upregulated in plants sprayed with 40 mg L^−1^ of TiO_2_ NPs under biotic stress (Tables S1–S4), which are mainly associated with oxidative burst after fungal invasion. The upregulation of these proteins reported in this study is in accordance with the results shown by the interaction of barley and powdery mildew [41]. The enhanced production of various antioxidant proteins in the infected leaves indicate oxidative burst [43]. ROS accumulation more rapidly occurs in resistant than susceptible plants during pathogen infections. Barna et al. [44] suggested that the upregulation of antioxidants in infected plants can inhibit the pathogen-induced accumulation of ROS. The high level of antioxidants also suggests the association of plants and pathogens to protect the pathogen against oxidative damage [43].

When a plant recognizes an invasion or attack by a pathogen, the usual response places a huge demand on energy in plant cells so that the defense mechanisms within the plant body can be triggered and activated [45]. The carbohydrate metabolic pathways not only meets the demanding cellular energy requirements to trigger the defense system of plants, but also enhances the expressions of genes related to defense during the pathogen–plant interactions [45]. Most of the proteins identified in infected leaves were mainly associated with carbohydrate metabolism, and showed upregulation in fungal-pathogen-infected leaves in comparison with the control (Tables S1 and S2). The identified upregulated proteins were mainly associated with glycolytic pathways, Kreb’s cycle, and the pentose-phosphate pathway. The results also revealed that the fungal infection altered the patterns of gene expressions associated with the metabolism of carbohydrates and the elevated production of sugars, such as pentose or hexoses, which act as metabolic signals to activate the plant defense system [2,43,46]. The data showed the upregulation of the enzyme 6-phosphogluconate dehydrogenase in pathogen-infected leaves. This enzyme is involved in various reactions of the pentose-phosphate pathways to produce NADPH, which in turn is involved in facilitating the activity of NADPH-oxidase, which is the main ROS-producing enzyme during infection by pathogens [21,43,44].

The results of proteome analysis revealed the expression of many proteins associated with the tricarboxylic acid (TCA) cycle, such as iso-citrate dehydrogenase, in the infected plants treated with 40 mg L^−1^ of TiO_2_ NPs (Tables S3 and S4). The TCA cycle plays an important role in the defense responses of plants, as isocitrate dehydrogenase is involved in producing NADH to trigger the redox signaling associated with pathogen responses [47]. The data also revealed the accumulation of respiration-associated proteins, such as malic enzyme, which was upregulated in infected plants with exposure to the same treatment (Table S2). Similar findings were reported by [43], who found NADP-dependent malic enzyme was upregulated in resistant wheat varieties during plant–pathogen interactions. The upregulation of carbohydrate-associated proteins in pathogen-infected wheat leaves also led to the speculation that these may be involved in helping plants to combat stress and triggering defense responses [45].

The results also showed a downregulation of proteins associated with photosynthesis in fungal-pathogen-infected wheat leaves (Table S2). The findings of previous studies have also indicated that the photosynthetic rate declined after the invasion by the pathogen [21,45,48]. The downregulation of photosynthesis in infected leaves might have been due to the decline in Rubisco activase enzyme abundance as well as the downregulation of Rubisco large subunit binding protein in the infected wheat leaves. Similar findings were observed during the interaction of *Arabidopsis thaliana* with powdery mildew [45].

## 3. Materials and Methods

### 3.1. Preparation of Aqueous Plant Leaf Extract

Aqueous leaf extract of *Moringa oleifera* Lam. was prepared to fabricate the TiO_2_ NPs. Fresh leaves of *Moringa oleifera* were collected with the permission from the research coordinator (Dr. Zia-Ur-Rehman Mashwani), and were identified by Dr. Naveed Iqbal Raja (Department of Botany, PMAS Arid Agriculture University, Rawalpindi, Pakistan). The specimen voucher (SHS-001) was prepared and deposited in the herbarium of the Department of Botany, PMAS-Arid Agriculture University. Plant leaves were thoroughly washed using distilled water to remove soil and dust particles. Approximately 50 g of leaf pieces was transferred into a beaker containing distilled water (400 mL) and subjected to boiling for 20 min. The extract was filtered three times with Whatman No. 1 filter paper (GE Healthcare Bio-Sciences, Pittsburgh, PA, USA), and used for the reduction of the Ti(OH)_2_ solution [49,50].

### 3.2. Biogenesis of TiO_2_ NPs

The green synthesis of titanium dioxide nanoparticles was carried out by adding 10 mL of plant extract of *M. oleifera* in a 5 mM solution of titanium dioxide salt under stirring conditions at 50 °C and 1.5 pH. The reaction mixture color changed from white to pinkish-brown, indicating TiO_2_ NPs synthesis. The solution mixture was centrifugated at 10,000 rpm for 20 min at room temperature. The resultant pellet was dried via a SpeedVac concentrator (SPD 120, Thermo Fisher Scientific, Waltham, MA, USA) and used for further experiments [12].

### 3.3. Physical and Optical Characterization of TiO_2_ NPs

#### 3.3.1. UV–Visible Spectrophotometry

Reduction of TiO_2_ salt solution into TiO_2_ NPs using the aqueous extract was initially confirmed by measuring the UV–Visible spectrum (Labomed UVD 3500, Los Angeles, CA, USA) ranging in wavelength from 200 to 900 nm [51].

#### 3.3.2. Energy-Dispersive X-ray Spectroscopic Analysis (EDX Analysis)

The green-synthesized TiO_2_ NPs were evaluated for elemental composition through EDX analysis. The sample was prepared by dropping the green synthesized TiO_2_ NPs on a thin film of carbon, as described by Javed et al. [52].

#### 3.3.3. Scanning Electron Microscopic Analysis (SEM Analysis)

The morphological structure of the biosynthesized TiO_2_ NPs was studied using SEM. An SEM SIGMA (TESCAN MIRA3 FEG-SEM, NanoImages, LLC, Pleasanton, CA, USA) was used at 5 kV along with magnification of 10,000×. A minor amount of the prepared sample was dropped on a carbon-coated grid. The films were allowed to dry using a lamp of mercury for 7 min, and the excessed solution was removed with blotting paper [53].

#### 3.3.4. Fourier Transform Infrared Analysis (FTIR Analysis)

FTIR analysis was performed to identify the functional groups contributing to the reduction and stabilization of the biogenic TiO_2_ NPs. The dried TiO_2_ NP powder was mixed with KBr pellets in a ratio of 1:100. The resulting powder mixture was turned into a pellet via a hydraulic apparatus. The spectrum was recorded in the range of 450 to 3900 cm using a Perkin-Elmer spectrum (Perkin-Elmer FTIR-Spectrum, Akron, OH, USA) [49].

### 3.4. Preparation of Fungal Inoculum 

The urediniospore of *Pst* (strain 572432) was collected from the Crop Disease Research Institute of the NARC, Islamabad. In distilled water, the urediniospores were suspended, and 1 mg L^−1^ Tween 20 was used as a surfactant. Approximately 0.6 mL of spore suspension was prepared per plant at the rate of 6 × 10^5^ spores/mL. The concentration of the spores in the inoculum was determined using a hemocytometer by placing 20 µL of inoculum on the grid. Spores that appeared pale in color or deformed were separated. The bright yellow and spherical spores were selected as healthy and viable [12].

### 3.5. Preparation of Greenhouse Experiments and Treatment Plan

A greenhouse experiment was conducted to evaluate the antifungal efficacy of the green-synthesized TiO_2_ NPs on wheat plants against *Pst*. Wheat plants were grown in pots (24 cm in diameter and 19 cm in height) filled with sandy loam soil (sand: 44.2%, silt: 4.6%, and clay: 51.2%). The greenhouse environmental conditions were sustained between 21 and 25 °C, with relative humidity between 80% and 90%. Illumination was supplemented for 16 h/day with fluorescent lights. The fungus-susceptible wheat variety (Galaxy-2013) was collected from the NARC, Islamabad. Seeds were surface-sterilized with 0.1of mercuric chloride for 3 min, and five to eight seeds were sown into each pot. The experiment design was a completely randomized block design with three replicates. To elucidate the effect of biosynthesized TiO_2_ NPs on wheat plants against yellow rust, various concentrations of TiO_2_ NPs were prepared. A stock solution of TiO_2_ NPs (100 mL) was prepared by adding 0.5 g of the biosynthesized TiO_2_ NPs powder into 100 mL of distilled water. The resultant nanoparticle solution was subjected to ultrasonication at high amplitude to enhance TiO_2_ NP stability and homogeneity in the aqueous solution. Various concentrations (20, 40, 60, and 80 mg L^−1^) were designed by diluting the stock solution to evaluate the effect of the nanoparticles in combating fungus growth in comparison with control plants [12]. The detailed treatment plan is given in Table 1.

### 3.6. Inoculation of Wheat Plants with Pst

Wheat plants were grown until the two-leaf stage. The plants were given a cold treatment at night, and the temperature was maintained at 4 °C. The day temperature was maintained below 25 °C for 15 days. The plants were kept in a dew chamber, and water and wall chamber temperatures were adjusted. The air temperature was adjusted to 15 °C, which is the optimum temperature for yellow rust growth, achieving dew formation by setting the water to 20 °C and the wall to 5 °C. These conditions were set for 1 h before placing the wheat plants in the chambers. Distilled water was used to minimize salt precipitation and trace minerals produced on heating elements, which can reduce the germination and growth of yellow rust spores. The inoculum bottle was attached to the propellant, and inoculum was sprayed on front and back of the plant from 5 inches away with one hand behind the plants to catch most of the inoculum. The dew chamber was incubated for 48 h. The plants were then moved to a greenhouse, and the growing conditions were adjusted to a 16 h photoperiod with a night temperature of 4 °C and a day temperature below 25 °C until the onset of disease [54,55].

### 3.7. Foliar Application of Biosynthesized TiO_2_ NPs

Wheat plants were sprayed with different concentrations (20, 40, 60, and 80 mg L^−1^) of the biosynthesized TiO_2_ NPs three times each. The first foliar spray was applied at the two-leaf stage before the inoculation of *Pst* spores. The second and third sprays were applied at the tillering and flag leaf stages, respectively.

### 3.8. Assessment of Disease Severity

Leaf tissues were randomly obtained from selected plants in every treatment. The first assessment was performed 10 days after the inoculation, and the same process was repeated every 10th day until the plants were harvested. The symptoms indicating the severity of stripe rust were evaluated with the help of a 0 to 5 rating scale on a visual basis (Table 2) [12].

### 3.9. Assessment of Disease Incidence

Yellow rust disease incidence was measured using the following formula [55]:

(1) $$\mathrm{Disease}\;\mathrm{Incidence}\;\left(\%\right)=\frac{\mathrm{Number}\;\mathrm{of}\;\mathrm{infected}\;\mathrm{plants}}{\mathrm{Total}\;\mathrm{number}\;\mathrm{of}\;\mathrm{plants}}\times 100$$

The percent disease index was determined using the following formula [56]

(2) $$\mathrm{Percent}\;\mathrm{Disease}\;\mathrm{Index}\;\left(\mathrm{PDI}\right)=\frac{\mathrm{Disease}\;\mathrm{index}}{\mathrm{Total}\;\mathrm{infected}\;\mathrm{plants}}\times 100$$

Disease index = (Stripes in scale 1) + (Stripes in scale 2) + (Stripes in scale 3) + (Stripes in scale 4) + (Stripes in scale 5)(3)

### 3.10. Measurement of Biochemical Attributes of Wheat Plants Treated with TiO_2_ NPs

#### 3.10.1. Determination of Proline Content

Proline content was measured following the protocol of Bates et al. [57], with slight modifications. Approximately 250 mg of wheat plants leaf sample was homogenized in 5 mL of sulfosalicylic acid (3%). We transferred 2 mL of the resultant filtrate into separate test tubes, which we mixed with 2.5 mL of ninhydrin reagent and 2.5 mL of acetic acid. After boiling, the reaction was immediately stopped after the color development by placing the test tubes on ice. Furthermore, 4.5 mL of toluene was added to the resultant mixture and properly stirred until a colored layer appeared on the surface. This layer was separated and placed in another test tube. The absorbance of the sample was observed at a 550 nm wavelength. The proline content was determined with the following formula:

Total proline (µg mL^−1^) = (Sample absorbance × Dilution factor × K value plant tissue fresh weight) (4)

#### 3.10.2. Determination of Total Soluble Phenolic Content

The total phenolic content of leaves was evaluated following the protocol described by Abd Allah et al. [18]. Fresh leaves (0.8 g) were homogenized with 15 mL of methanol and left overnight. The extract was filtered, diluted with 100 mL of water, and used as a stock solution. Thereafter, approximately 250 µL of the aqueous extract was added to 1.5 mL of distilled water and 0.9 mL of 50% Folin–Ciocalteu phenol reagent [58]. The mixture was placed to rest for 3 min, followed by the addition 0.6 mL of 23% (*w*/*v*) sodium carbonate (0.4%). The resultant solution was vortexed after 2.5 h. The absorbance was then recorded at 765 nm using gallic acid as a standard.

#### 3.10.3. Measurement of Total Flavonoid Content

The total flavonoid content was determined with some modifications [59]. Approximately 500 mg of leaves was crushed and mixed with methanol (85%). The mixture was centrifuged at 25,000 rpm for 15 min at 25 °C. The supernatant was mixed with 5 mL distilled water, followed by 0.7 mL of 5% sodium nitrate and 0.6 mL of 15% aluminum chloride. The resulting mixture was kept for 5 min at room temperature. The solution was thoroughly mixed after adding 4 mL of 1 M sodium hydroxide and 3 mL of distilled water, and left once again for 1 min. The solution absorbance was documented at a wavelength of 510 nm. 

### 3.11. Antioxidant Enzymes Assay

#### 3.11.1. Preparation of Leaf Extract for Enzyme Assays

Wheat leaf extract was prepared to determine the antioxidant potential following the method reported by [60], with some modifications. Approximately 150 mg of fresh leaves was mixed with 15 mL of extraction buffer (10% polyvinylpyrrolidone and 0.1 M of ethylene diamine-tetra-acetic acid (EDTA)). The resulting solution was subjected to 10,000 rpm centrifugation for 15 min under cooling. The supernatant was further utilized in various enzyme assays. 

#### 3.11.2. Peroxidase Activity (POD)

The aforementioned leaf extract was used for the POD (EC 1.11.1.7) assay, following the method described by [61]. Enzyme extract (0.2 mL) was mixed with 1.35 mL of 0.2 M MES buffer (pH less than 5.6), 0.05% hydrogen peroxide, and 0.2% phenylenediamine. For each treatment, absorbance was recorded for 3 min at 485 nm.

#### 3.11.3. Superoxide Dismutase Activity (SOD)

The SOD (EC 1.15.1.1) activity was measured using the protocol reported by [62], with some modifications. Enzyme extract (600 µL) was added to 200 µL of 0.076 mM nitroblue tetrazolium (NBT), 25 µL of 0.04 mM of riboflavin, 250 µL of 1.5 mM EDTA, 250 µL of 135 mM methionine, and 800 µL of phosphate buffer. A blank was used for comparison with the reaction mixture. The blank was prepared by adding the same compounds in the reaction mixture except for the enzyme extract. The absorbance was recorded at 560 nm for both the reaction mixture and blank after 7 min of exposure to fluorescent light. The Beer-Lambert law was then applied to calculate the SOD activity:

A = ε LC(5)

where ε is the extinction coefficient, L is the length of the wall, and C is the concentration of the enzyme.

#### 3.11.4. Catalase Activity (CAT)

To determine the CAT (EC 1.11.1.6) activity, the protocol described by [63] was followed, with slight alterations. We prepared 200 mL of the reaction mixture by adding 500 µL of the enzyme extract in 250 µL of 28 mM hydrogen peroxide, 450 µL of phosphate buffer, and 950 µL of distilled water. The blank was prepared by adding same compounds except for the enzyme extract. The absorbance was recorded at 250 nm for both the reaction mixture and blank after 7 min of exposure to fluorescent light. 

### 3.12. Proteome Analysis of Wheat Plants under Biotic Stress

Three treatments (T_0_, T_1_, and T_3_) of wheat plant leaves were selected for the proteome analysis. T_0_ was the control, T_1_ was leaves under fungal stress but without TiO_2_ NP application, and T_3_ was leaves inoculated with the stripe rust disease and 40 mg L^−1^ of TiO_2_ NPs.

#### 3.12.1. Extraction of Proteins for Proteome Analysis

Flag leaves were harvested from the control and treated plants. The leaves were immediately placed in liquid nitrogen and stored at −80 °C until further use. The frozen tissues were ground to create the fine leaf powder using autoclaved and a prechilled mortar and pestle with liquid nitrogen. The ground leave powder was mixed with 25 mm of Tris-HCl (pH 8), 4% of CHAPS, 5.5 mM of EDTA, and 2.5 mM of phenyl-methyl-sulfonyl fluoride. The mixture was gently shaken to form a fine solution. The formed solution was poured into 2 mL Eppendorf tubes and centrifuged at 20,000 rpm for 20 min under cooling. The supernatant was mixed with 4 volumes of ice-cold acetone solution containing 10% of trichloroacetic acid and 0.07% of 2-mercapto-ethanol [21].

This procedure resulted in the precipitation of proteins present in the supernatant. The resultant mixture was subjected to vortexing and sonication for 15 min, and was then placed into an incubator for 1 h at −20 °C. The mixture was centrifuged at 10,000 rpm for 20 min at 4 °C. The supernatant was decanted, and the pellet was collected and thoroughly washed using 0.07% 2-mercaptoethanol mixed with acetone. After washing, the pellet was dried using a SpeedVac concentrator (SPD 120, Thermo Fisher Scientific, Waltham, MA, USA), and suspended again in lysis buffer containing 6.5 M of urea, 2 M of thiourea, 5% of CHAPS, and 2 mM of tri-butyl-phosphine. The mixture was vortexed for 60 min at room temperature. The formed suspension after vortex was centrifuged twice at 20,000 rpm for 20 min at room temperature, and the supernatant was collected as the total proteins. The concentration of the proteins in the supernatant was calculated using a Bradford assay, where bovine serum albumin was used as the standard [64].

#### 3.12.2. Resuspension, Denaturing, Reduction, Alkylation, and Digestion of Proteins for Mass Spectrometric Analysis

The protein samples were purified using methanol and chloroform to retain detergent-free protein samples [21]. The total protein (0.5 mg) was added to 0.5 mL Eppendorf tube. We added 25 µL of ammonium bicarbonate solution and 25 µL of trifluoroethanol (TFE) denaturation agent to the mixture. We added 1.0 µL of dithiothreitol (DTT) stock solution, which we placed on the vortex to mix. After vortexing, the solution was subjected to heating for 45 min at 60 °C, and left to cool at room temperature. The solution was alkylated using 4 µL of 0.5 M iodoacetamide (IAM) stock solution, briefly vortexed and incubated in the dark at room temperature for one hour. After incubation, 300 µL of water was added to dilute the denaturant. The pH was raised between 7 and 9 using 100 µL of ammonium bicarbonate stock solution. Proteins were digested using trypsin. Trypsin was added to the protein solution at a 1:20 mass of enzyme:substrate ratio, that is, 10 to 25 µL of trypsin. The solution was vortexed, placed on a heater, incubated at 37 °C for 4 to 18 h, and then subjected to cooling. Neat formic acid (1 µL) was added to lower the pH and stop the trypsin activity. The samples were diluted to achieve a 50 fmol µL^−1^ solution. This solution was used for the analysis of nano-liquid chromatography/mass spectroscopy (LC/MS).

#### 3.12.3. Identification of Proteins through Nano-Liquid Chromatography Mass Spectrometry

The peptides were dissolved in 0.1% of formic acid and loaded into an Ultimate 3000 Nano LC/MS system (Dionex, Germering, Germany) containing a C18 PepMap trap column (300 µm × 5 mm, Dionex). The peptides were removed from the trap column, and then separated by adding 0.1% of formic acid in a linear gradient of acetonitrile at a flow rate of 200 µL min^−1^ on a C18 Tip column (75 µm of ID × 120 mm; Nikkyo Technos, Tokyo, Japan) and a spray voltage of 2 kV. Peptides were detected and analyzed using a nanospray LTQ Orbitrap elite MS (Thermo Fisher Scientific, Waltham, MA, USA). The mass spectrum was obtained in the MS range of 400–1500 *m*/*z* with a resolution of 40,000. High mass accuracy was obtained through a lock mass function [65]. The ten most vigorous precursor ions were chosen for collision-generated fragmentation in the linear ion trap at a normalized energy of collision 35.5% with an activation time of 40,000; dynamic exclusion was applied within 90 s to avoid repetition in the peptides [41].

#### 3.12.4. Identification of Proteins from the Mass Spectrometry Data

The proteins were identified by the Mascot search engine (version 2.5.1; Matrix Science, London, UK) and UniProt Knowledgebase SwissProt (http://www.uniprot.org, accessed on 1 May 2022). From the raw data files, the DTA files were formed and changed to Mascot generic files using Proteome Discoverer software (version 1.4.0.289; Thermo Fisher Scientific, Waltham, MA, USA). Carbamido-methylation of cysteine was established as fixed modification, and methionine oxidation was established as variable modification in the Mascot searches. Moreover, the trypsin was fixed as the digestive enzyme, and one missed cleavage was allowed. The peptide mass tolerance was fixed to 9 mg L^−1^, and fragment mass tolerance was fixed to 0.9 Da. Mascot percolator was used to filter the Mascot results for improving the precision and sensitivity of peptide identification [66]. For all searches, the false discovery rates of the peptide identification were less than 1%. Peptides having a percolator ion score greater than 15 (*p* < 0.05) were utilized.

#### 3.12.5. Differential Analysis of Identified Proteins

Using MSF format, the Mascot search results were exported and subjected to SIEVE analysis (version 2.1; Thermo Fisher Scientific, Waltham, MA, USA). The abundance of and change in proteins between the control and treated groups were performed with the help of SIEVE. The MS detected the chromatographic peaks for analysis. The peaks were detected, aligned, and used to further detect the q peaks in the form of the frame. The frame width time was set to 5 min, and the *m*/*z* width of the frame was set to 10 mg L^−1^. These parameters were used to form the frames for every parent ion scanned using MS/MS. The comparative analysis of the chromatographic peak area of all samples was conducted, and the ratios of control and treated groups were determined in each frame. The frames sorted by MS/MS scanning were matched with the Mascot search results. The ratio of the control- to the treated-group peptides was found by the variance weighted average of the ratios determined in the frames, matching the peptides in the MS/MS spectrum. The determined ratios of the peptides were integrated to find the ratios of the related proteins. The required minimum parameter to analyze the identification of the proteins was at least 2 matched peptides. The significant differences in the frequency of proteins between the control and treated groups were determined (*p* < 0.05). The protein content was determined using molar fraction percentage (molar %).

### 3.13. Bioinformatics Analysis

The functional role of the identified proteins by MS analysis was determined using the UniProt IDs (https://www.uniprot.org, accessed on 1 May 2022), which was changed to TAIR IDs using ID conversions (https://www.arabidopsis.org, accessed on 1 May 2022). The functional categorization of the proteins was performed using MapMan bin codes [67]. The selected stress- and defense-related genes were subjected to STRING software (https://string-db.org, accessed on 1 May 2022) to determine the correlation between the gene of interest to other proteins and their involvement in various metabolic pathways.

### 3.14. Statistical Analysis

The experiment design was a completely randomized block design with three replicates (blocks). The pots were arranged in whole-plot groups. The greenhouse and blocks were considered random factors. To determine the significant differences between control and treated groups, statistical analyses using Duncan’s multiple range test (*p* < 0.05) were performed. SPSS software (version 18; Armonk, New York, NY, USA) was employed to perform calculations.

## 4. Conclusions

In this study, we focused on the biogenic synthesis of TiO_2_ NPs from the aqueous leaf extract of *Moringa oleifera*. The results of the application of various structural and optical analytical techniques showed that the nanoparticles were in the size range of 10–100 nm and spherical and irregular in shape. We also confirmed that the biochemical functional groups consist of O-H, N-H, C-C, and Ti-O stretching, which are responsible for the stabilization and biochemical potential of the nanoparticles. The exogenous applications of various concentrations of nanoparticles on wheat plants resulted in the reductions in disease incidence and percentage disease index, with the highest pathogenic inhibition observed for treatment with 40 mg L^−1^ of TiO_2_ NPs. The results of differential proteome analysis of infected wheat plants and plants treated with the 40 mg L^−1^ of TiO_2_ NPs confirmed that the up- and downregulation of proteins were the main forces triggering the defense-related responses in wheat plants. The results of differential protein analysis confirmed that the response to biotic stress in wheat plants has energy requirements that the plant meets by maintaining a balance by enhancing the activities of the carbohydrate metabolic pathways. The findings confirm the promising role of biogenic TiO_2_ NPs in triggering the up- and downregulation of proteins that enhance defense and disease resistance in wheat plants against the biotic stress imposed by *Puccinia striiformis* f. sp. *tritici* (*Pst*).

## Figures and Tables

**Table 1:** Treatment plan and experimental conditions.

Treatment	Condition
T_0_	Control (healthy wheat plants)
T_1_	Plants infected with pathogen (*Puccinia striiformis*) only
T_2_	Plants infected with pathogen + 20 mg L^−1^ TiO_2_ NPs
T_3_	Plants infected with pathogen + 40 mg L^−1^ TiO_2_ NPs
T_4_	Plants infected with pathogen + 60 mg L^−1^ TiO_2_ NPs
T_5_	Plants infected with pathogen + 80 mg L^−1^ TiO_2_ NPs

**Table 2:** The rating scale for yellow rust evaluation of infected wheat plants.

Number	Level of Symptoms	Resistance Level
0	No symptoms	Resistant
1	1–5% stripes on leaves	Moderately resistant
2	6–20% stripes on leaves	Moderately resistant
3	21–40% stripes on leaves	Moderately susceptible
4	41–60% stripes on leaves	Moderately susceptible
5	More than 61% stripes on leaves	Susceptible

## Data Availability

All obtained data are presented in this article and Supplementary Materials.

## Supplementary Materials

The following supporting information can be downloaded at: https://www.mdpi.com/article/10.3390/molecules27134274/s1, Table S1: Relative abundance and functional categorization of upregulated proteins of wheat plants under *Puccinia striiformis* stress; Table S2: Relative abundance and functional categorization of downregulated proteins in wheat plants under *Puccinia striiformis* stress; Table S3: Relative abundance and functional categorization of upregulated proteins in response to 40 mg L^−1^ of TiO_2_ NPs under *Puccinia striiformis* stress; Table S4: Relative abundance and functional categorization of downregulated proteins of wheat plants treated with 40 mg L^−1^ of TiO_2_ NPs for *Puccinia striiformis* infection.

## Sample Availability

Not available.
