# Supplementary material for: Plant-Based Titanium Dioxide Nanoparticles Trigger Biochemical and Proteome Modifications in Triticum aestivum L. under Biotic Stress of Puccinia striiformis
Source: Molecules. 2022 Jul 2;27(13):4274. doi: 10.3390/molecules27134274 (PMC9268011; doi:10.3390/molecules27134274)
Supplement: Supplementary file 1 [file molecules-27-04274-s001.zip › molecules-1780421-supplementary.pdf]

Supplementary Materials

Phyto-fabricated titanium dioxide nanoparticles mediated biochemical and proteome modifications in *Tritium aestivum* L. under the biotic stress of *Puccinia striiformis*

Seema Hassan Satti [1], Naveed Iqbal Raja [1], Muhammad Ikram [1], Ahmad A. Omar [2, 3*], Zia-Ur-Rehman Mashwani [1], Azza H. Mohamed [3, 4], Hesham Oraby [5, 6], Ajit Singh [7]

Table S1. Relative abundance and functional categorization of up-regulated proteins of wheat plants under *Puccinia striiformis* stress.

No.	Protein IDs	Species	Description	Normalized Average (TIL) mg.L^{-1}	Normalized Average (T0L) mg.L^{-1}	P Value	Functions
1	A0A1D6BFP3	*Triticum aestivum*	Bifunctional protein FolD	249.395	237.872	0.04	Amino acid biosynthesis
2	M8CE78	*Aegilops tauschii*	Actin-1	289.734	269.741	0.05	ATP binding
3	M8D3R3	*Aegilops tauschii*	Histone H4	123.803	77.536	0.03	DNA binding
4	A0A1D6BMJ7	*Triticum aestivum*	Fructose-bisphosphate aldolase	159.659	136.067	0.01	DNA binding
5	M8BLC1	*Aegilops tauschii*	Uncharacterized protein	197.264	155.131	0.01	DNA binding
6	A0A2A4UD83	*Moraxellaceae bacterium*	DNA polymerase III subunit epsilon	257.880	202.672	0.02	DNA replication
7	A0A1D5SSB2	*Triticum aestivum*	Uncharacterized protein	270.461	267.300	0.04	Enzymatic activity
8	M8C9E4	*Aegilops tauschii*	Fructose-bisphosphate aldolase	373.147	319.749	0.03	Glycolytic process
9	A0A2G1ZD24	*Shewanella sp*	Fructose-1,6-bisphosphate aldolase	288.645	257.614	0.04	Glycolytic process
10	D5WHJ9	*Triticum aestivum*	Diguanylate cyclase	321.892	300.673	0.02	Integral component of membrane
11	A0A1D6RID2	*Triticum aestivum*	RING finger protein 175	458.473	455.782	0.02	Metal-ion binding
12	A0A341SDD9	*Triticum aestivum*	Uncharacterized protein	387.740	355.061	0.03	Not assigned
13	A0A341R419	*Triticum aestivum*	Uncharacterized protein	351.307	317.292	0.01	Not assigned
14	A0A341RJ55	*Triticum aestivum*	Uncharacterized protein	278.162	219.907	0.01	Not assigned
15	A0A341WRC8	*Triticum aestivum*	Uncharacterized protein	378.039	321.184	0.03	Not assigned
16	A0A1D6RID4	*Triticum aestivum*	Uncharacterized protein	461.276	412.560	0.02	Not assigned
17	A0A1D6REJ3	*Triticum aestivum*	Uncharacterized protein	377.209	361.158	0.04	Not assigned
18	W5GSM3	*Triticum aestivum*	Uncharacterized protein	256.464	219.671	0.01	Not assigned
19	A0A341Y173	*Triticum aestivum*	Uncharacterized protein	347.789	318.837	0.04	Not assigned
20	A0A1D5SAJ4	*Triticum aestivum*	Uncharacterized protein	389.262	313.004	0.02	Not assigned
21	A0A341PU48	*Triticum aestivum*	Uncharacterized protein	275.783	216.473	0.01	Not assigned

22	W5BGE9	*Triticum aestivum*	Uncharacterized protein	219.529	184.389	0.01	Not assigned
23	ǀA0A1D5TH95	*Triticum aestivum*	Uncharacterized protein	167.568	156.711	0.02	Not assigned
24	A0A341W521	*Triticum aestivum*	Uncharacterized protein	281.760	277.568	0.04	Not assigned
25	A0A341Q4P7	*Triticum aestivum*	Uncharacterized protein	383.832	356.729	0.03	Photosynthesis
26	A0A220IKU8	*Triticum turgidum*	Cytochrome b6 (Fragment)	251.725	247.884	0.03	Photosynthesis
27	S17P	*Arabidopsis thaliana*	Sedoheptulose-1,7-bisphosphatase, chloroplastic	178.672	157.047	0.01	Plant Defense
28	S4SD74	*Aegilops speltoides*	Ribulose bisphosphate carboxylase large chain	390.371	326.369	0.02	Respiration
29	R7W1Q1	*Aegilops tauschii*	Malate dehydrogenase	327.668	277.462	0.04	Respiration
30	M8C9D5	*Aegilops tauschii*	Ribonuclease 1	384.504	372.970	0.03	RNA binding
31	A0A1D5WDE3	*Triticum aestivum*	Uncharacterized protein	256.667	212.681	0.05	Stress response
32	A0A1D5WDE2	*Triticum aestivum*	Uncharacterized protein	299.370	277.162	0.04	Stress response
33	A0A1B5GE54	*Triticum aestivum*	Caleosin	217.020	166.793	0.03	Stress response
34	E7D433	*Triticum aestivum*	EF-hand calcium-binding protein	288.952	266.192	0.02	Stress response
35	M8A9K0	*Triticum urartu*	Uncharacterized protein	357.684	331.588	0.04	Stress response
36	G8IS39	*Pinus yunnanensis*	Photosystem II protein D1	337.705	289.053	0.01	Stress response
37	M7YPJ9	*Triticum urartu*	14 kDa proline-rich protein DC2.15	464.582	455.179	0.01	Stress response
38	H9NAV2	*Triticum aestivum*	Chloroplast Cu/Zn superoxide dismutase (Fragment)	335.893	310.050	0.01	Stress response
39	A0A1D6CM42	*Triticum aestivum*	Superoxide dismutase [Cu-Zn]	147.451	137.672	0.01	Stress response
40	M8BN49	*Aegilops tauschii*	50S ribosomal protein L12-2, chloroplastic	480.787	417.495	0.02	Translation
41	A0A1D5WKK3	*Triticum aestivum*	Uncharacterized protein	375.690	372.654	0.04	Transport
42	1D5WKK4	*Triticum aestivum*	Uncharacterized protein	256.538	227.629	0.03	Transport

Table S2. Relative abundance and functional categorization of down-regulated proteins in wheat plants under *Puccinia striiformis* stress.

No.	Protein IDs	Species	Description	Normalized Average (TIL) mg.L^{-1}	Normalized Average (T0L) mg.L^{-1}	P-Value	Functions
1	A0A1D5U3N1	*Triticum aestivum*	Uncharacterized protein	276.183	286.739	0.01	Arginine and proline metabolism
2	A0A218M001	*Triticum turgidum*	ATP synthase subunit b, chloroplastic	289.679	307.298	0.03	ATP synthesis
3	A0A077RST2	*Triticum aestivum*	Carbonic anhydrase	378.184	394.647	0.02	carbon utilization
4	A0A1D5UJL4	*Triticum aestivum*	Uncharacterized protein	162.178	236.165	0.02	Catalytic activity
5	A0A1D6D889	*Triticum aestivum*	Thioredoxin	219.047	233.312	0.04	Defense response
6	D8L9G3	*Triticum aestivum*	Sedoheptulose-1,7-bisphosphatase, chloroplast, expressed	337.275	372.183	0.02	Defense response
7	M8B2C7	*Triticum aestivum*	Sedoheptulose-1,7-bisphosphatase, chloroplastic	72.196	86.066	0.01	Defense response
8	A0A1D6RID7	*Triticum aestivum*	Lymphoid enhancer-binding factor 1	274.190	287.179	0.01	Defense response
9	M8BLC1	*Aegilops tauschii*	Uncharacterized protein	223.891	247.490	0.05	DNA binding
10	T1L8T4	*Triticum urartu*	Histone H4	437.372	488.146	0.04	DNA binding
11	A0A2A4TTB9	*Elusim icrobia bacterium*	Uncharacterized protein	383.787	402.378	0.03	DNA binding

12	M8C4F9	*Aegilops tauschii*	Plastocyanin	253.183	277.392	0.02	Electron transfer activity
13	A0A1D5SZE5	*Triticum aestivum*	Uncharacterized protein	267.752	288.103	0.04	Enzymatic activity
14	Q0WXG8	*Triticum aestivum*	Triticain alpha	212.005	240.878	0.01	Growth regulation
15	A0A2X0SW30	*Triticum aestivum*	Uncharacterized protein	177.832	201.371	0.03	Growth regulation
16	A0A1D6C852	*Triticum aestivum*	Fructose-bisphosphate aldolase	194.749	237.163	0.01	Host cell surface receptor binding
17	Q6LBU2	*Triticum aestivum*	RbcL gene product (30 AA) (Fragment)	123.847	167.940	0.05	Magnesium ion-binding
18	A0A2G2MTL5	*Robigi nitomaculum*	Formate dehydrogenase	244.926	278.199	0.04	Metal-ion binding
19	A0A1D6RID6	*Triticum aestivum*	E3 ubiquitin-protein transferase MAEA	303.192	384.254	0.04	Negative regulation of gluconeogenesis
20	A0A341SEH0	*Triticum aestivum*	Uncharacterized protein	357.281	384.269	0.02	Not assigned
21	A0A341QH30	*Triticum aestivum*	Uncharacterized protein	69.736	73.273	0.01	Not assigned
22	A0A1D5S0I6	*Triticum aestivum*	Uncharacterized protein	156.934	275.294	0.04	Not assigned
23	W4ZRQ6	*Triticum aestivum*	Uncharacterized protein	177.582	204.285	0.03	Not assigned
24	A0A1D5YGN4	*Triticum aestivum*	Uncharacterized protein	243.186	274.678	0.02	Not assigned
25	A0A1D5Z536	*Triticum aestivum*	Uncharacterized protein	378.199	384.385	0.04	Not assigned
26	A0A341PKR8	*Triticum aestivum*	Uncharacterized protein	457.560	488.211	0.01	Not assigned
27	W5ASV0	*Triticum aestivum*	Uncharacterized protein	164.893	176.037	0.01	Not assigned

28	W5EGN1	*Triticum aestivum*	Uncharacterized protein	267.870	289.479	0.01	Not assigned
29	A0A1D5YP02	*Triticum aestivum*	Uncharacterized protein	137.951	174.835	0.04	Not assigned
30	A0A341U9K6	*Triticum aestivum*	Uncharacterized protein	401.059	463.784	0.02	Not assigned
31	M8AE10	*Triticum urartu*	Oxygen-evolving enhancer protein 1, chloroplastic	255.985	371.381	0.03	Photosynthesis
32	W5AQE7	*Triticum aestivum*	Uncharacterized protein	278.472	305.386	0.01	Photosynthesis
33	N1R4X3	*Aegilops tauschii*	Oxygen-evolving enhancer protein 1, chloroplastic	337.410	373.193	0.03	Photosynthesis
34	M7ZWU9	*Triticum urartu*	Chlorophyll a-b binding protein, chloroplastic	398.139	404.740	0.02	Photosynthesis
35	Q2L3E3	*Brachy podium sylvaticum*	Photosystem 1 subunit 5	143.573	187.574	0.05	Photosynthesis
36	G8J4D7	*Pinus dalatensis*	Photosystem II protein D1 (Fragment)	260.649	278.163	0.04	Photosynthesis
37	A0A218M004	*Triticum turgidum*	Photosystem II protein D1	281.291	284.472	0.03	Photosynthesis
38	A0A218LW49	*Triticum aestivum*	Photosystem II protein D1 (Fragment)	307.183	338.748	0.03	Photosynthesis
39	A0A0W7VKQ7	*Trichode rmagamsii*	Uncharacterized protein	294.575	308.173	0.02	Protein disulfide oxidoreductase activity
40	Q6XPZ6	*Triticum aestivum*	Peptidyl-prolyl cis-trans isomerase	183.193	188.890	0.01	Protein folding
41	A0A1D5V1U8	*Triticum aestivum*	Cytochrome b6-f complex iron-sulf	327.782	389.137	0.04	Protein metabolism

			ur subunit				
42	A0A1D5ZYK7	*Triticum aestivum*	Uncharacterized protein	251.197	277.281	0.05	Protein-protein interaction
43	A0A2G2KMZ9	*Halomonas sp.*	Histidine kinase	278.912	304.338	0.03	Signaling
45	W5GHG3	*Triticum aestivum*	Caleosin	289.037	301.759	0.01	Stress response
46	M8BMC6	*Aegilops tauschii*	Putative L-ascorbate peroxidase, chloroplastic	337.173	364.833	0.02	Stress response
47	G8J0W2	*Pinus hwangshanensis*	Photosystem II protein D1	317.389	374.429	0.04	Stress response
48	C3SAB3	*Brachypodium distachyon*	Proline-rich protein	388.648	403.462	0.03	Stress response
49	A0A2W1CYE9	*Pyrenophora tritici-repentis*	Cell surface protein (Mas1) (Fragment)	153.902	167.419	0.02	Structural molecule activity
50	A0A2H3GU83	*Gibber ellazeae*	Uncharacterized protein	189.193	224.831	0.03	Transcription
51	T1MLC6	*Triticum aestivum*	Uncharacterized protein	265.479	278.194	0.04	Translation
52	M8A946	*Triticum urartu*	50S ribosomal protein L12-1, chloroplastic	201.104	247.193	0.02	Translation
53	M8C9E4	*Aegilops tauschii*	Fructose-bisphosphate aldolase	381.848	422.474	0.01	Glycolytic process

Table S3. Relative abundance and functional categorization of up-regulated proteins in response to 40 mg.L^{-1} of TiO2 NPs under *Puccinia striiformis* stress.

No.	Protein IDs	Species	Description	Normalized Average (T3L) mg.L^{-1}	Normalized Average (T0L) mg.L^{-1}	P Value	Functions
1	A0A1D5UEG2	*Triticum aestivum*	Acetyl-CoA acetyltransferase	378.671	319.894	0.04	Acetyl-co A-acetyltransferase activity
2	A0A1D6A8N9	*Triticum aestivum*	ABC transport system ATP binding protein	568.893	489.293	0.03	ATP binding
3	A0A2P4ZTZ1	*Trichoderma gamsii*	Mannose-1-phosphate guanylyltransferase	492.107	356.282	0.03	Biosynthetic process
4	A0A2G2L7J4	*Kangiella sp.*	Phosphoribulokinase	378.781	311.782	0.02	Carbohydrate metabolism
5	A0A341V0H6	*Triticum aestivum*	Uncharacterized protein	468.675	289.174	0.05	Carbohydrate metabolism
6	M8CYL1	*Aegilops tauschii*	Uncharacterized protein	367.278	117.812	0.03	Coenzyme binding
7	A0A1D6ARF6	*Triticum aestivum*	UDP-N-acetylmuramoylalanine--D-glutamate ligase	210.784	201.891	0.04	Degradation
8	R7W7A6	*Aegilops tauschii*	Histone H4	102.183	66.967	0.05	DNA Binding
9	A0A1D5U2B3	*Triticum aestivum*	Protein-L-isoaspartate O-methyltransferase	197.107	118.281	0.02	DNA repair
10	M8CY84	*Aegilops tauschii*	Ferredoxin-dependent glutamate synthase, chloroplastic	378.192	301.718	0.03	Glutamate biosynthetic process
11	A0A1D5UEF9	*Triticum aestivum*	Pyruvate formate-lyase-activating enzyme	532.178	309.285	0.01	Metal ion binding
12	A0A1D6B301	*Triticum aestivum*	Oxidoreductase molybdopterin binding	99.373	85.175	0.04	Nitrate assimilation
13	A0A1D5ZS58	*Trticum aestivum*	Uncharacterized protein	146.184	118.572	0.03	Not assigned
14	A0A1D5SGL1	*Trticum aestivum*	Uncharacterized protein	371.379	319.073	0.02	Not assigned

15	A0A1D5TP57	*Triticum aestivum*	Uncharacterized protein	467.927	300.963	0.01	Not assigned
16	O65795	*Trticum aestivum*	Histone H1	560.145	482.082	0.04	Nucleosome assembly
17	Q8L686	*Triticum aestivum*	Germin-like protein	290.216	210.728	0.03	Nutrient reservoir activity
18	M8D2E7	*Aegilops tauschii*	Germin-like protein 8-14	365.178	318.175	0.02	Nutrient reservoir activity
19	Q9M4V4	*Triticum aestivum*	Cytosolic glyceraldehyde-3-phosphate dehydrogenase GAPDH	147.738	119.967	0.02	Oxidoreductase activity
20	A0A1D6DBA5	*Triticum aestivum*	Membrane protease subunits, stomatin/prohibitin homologs	289.082	217.185	0.01	Peptidase activity
21	M7ZDA2	*Trticum urartu*	Flavoprotein wrbA	467.289	290.267	0.04	Photosynthesis
22	A0A218LW49	*Triticum aestivum*	Photosystem II protein D1 (Fragment)	529.995	412.784	0.03	Photosynthesis
23	M7ZCG5	*Triticum urartu*	Chlorophyll a-b binding protein, chloroplastic	504.182	389.035	0.03	Photosynthesis
24	A0A218LZA3	*Triticum turgidum*	Photosystem I P700 apoprotein A1	489.389	475.386	0.04	Photosynthesis
25	A0A075VYZ0	*Aegilops speltoides*	Photosystem I P700 chlorophyll a apoprotein A1	381.782	295.673	0.01	Photosynthesis
26	A0A075VVT5	*Aegilops speltoides*	Photosystem II D2 protein	290.671	218.083	0.05	Photosynthesis
27	M8C4F9	*Aegilops tauschii*	Plastocyanin	167.903	164.097	0.03	Photosynthesis
28	S4YZY7	*Triticum monococcum*	Photosystem I iron-sulfur center	390.174	371.659	0.02	Photosynthesis
29	A0A220IKU8	*Triticum turgidum*	Cytochrome b6 (Fragment)	291.782	219.740	0.03	Photosynthesis
30	A0A1D5SGL3	*Trticum aestivum*	Uncharacterized protein	119.637	78.192	0.01	Plant defense
31	G8IZV2	*Pinus pringlei*	Photosystem II protein D1 (Fragment)	58.963	22.891	0.04	Plant defense
32	M7Z200	*Triticum urartu*	ATP synthase delta chain, chloroplastic	489.672	431.489	0.02	Plant defense
33	Q7XYE2	*Triticum aestivum*	Catalase isozyme (Fragment)	374.863	318.674	0.04	Plant defense
34	M8C1W9	*Aegilops taushii*	L-ascorbate peroxidase 2, cytosolic	278.182	201.648	0.03	Plant defense
35	Q43219	*Triticum aestivum*	Peroxidase (Fragment)	290.673	217.783	0.04	Plant defense

36	A0A2A5D5U0	*Gammaproteobacteria bacterium*	Protein translocase subunit SecA	378.782	319.672	0.02	Protein targeting
37	A0A0D9B503	*Pseudomonas fluorescens*	Pyruvate carboxylase subunit B	163.361	161.387	0.03	Pyruvate carboxylase activity
38	M8CIQ4	*Aegilops tauschii*	Thioredoxin M-type, chloroplastic	178.572	166.834	0.01	redox
39	A0A1D6S409	*Triticum aestivum*	tRNA-dihydrouridine synthase	378.381	301.078	0.01	redox
40	M8B795	*Aegilops tauschii*	Ferredoxin--NADP reductase, chloroplastic	371.532	267.930	0.04	redox
41	M8CIQ4	*Aegilops tauschii*	Thioredoxin M-type, chloroplastic	457.196	401.749	0.03	redox
42	M8AQG0	*Triticum urartu*	Ribonuclease 1	518.924	499.056	0.02	RNA binding
43	M7ZVH4	*Triticum urartu*	31 kDa ribonucleoprotein, chloroplastic	469.386	318.174	0.04	RNA binding
44	O23798	*Triticum aestivum*	Ps16 protein	738.289	589.059	0.04	RNA binding
45	A0A1D6AKZ2	*Triticum aestivum*	Peptidase	494.896	412.068	0.05	Secondary metabolism
46	A0A1D5RRM4	*Triticum aestivum*	Phosphatidate cytidylyltransferase	389.117	385.682	0.03	Secondary metabolism
47	A6XMY5	*Trticum monococcum*	Serine hydroxymethyltransferase	183.863	182.087	0.02	Secondary metabolism
48	M7ZKR5	*Triticum urartu*	Receptor-like serine/threonine-protein kinase SD1-8	458.585	422.178	0.01	Signaling
49	A0A1B5GE54	*Triticum aestivum*	Caleosin	673.904	579.067	0.04	Stress response
50	N1QPF5	*Aegilops taushii*	Putative plastid-lipid-associated protein 3, chloroplastic	345.851	338.069	0.03	Stress response
51	A0A1D6BF30	*Triticum aestivum*	Acetyltransferase	438.696	437.008	0.05	Transferase activity
52	A0A1D6CM42	*Triticum aestivum*	30S ribosomal protein S13	237.680	217.704	0.03	Translation
53	M8BN49	*Aegilops tauschii*	50S ribosomal protein L12-2, chloroplastic	468.058	457.670	0.04	Translation
54	A0A1D6CI24	*Triticum aestivum*	Elongation factor EF-Tu	389.057	376.059	0.01	Translation
55	M8BN49	*Aegilops tauschii*	50S ribosomal protein L12-2,	177.705	152.057	0.01	Translation

			chloroplastic				
56	A0A1D6B0E1	*Triticum aestivum*	Transmembrane transporter	275.058	271.975	0.04	Transport
57	F9WY06	*Zymos eptoriatritici #*	Uncharacterized protein	286.056	172.067	0.03	Transport
58	A0A1D5WKK3	*Triticum aestivum*	Binding-protein-dependent transport systems	366.764	164.784	0.05	Transport
59	A0A1D6ABX5	*Triticum aestivum*	Two component transcriptional regulator	378.851	365.653	0.02	Transport
60	A0A2G2MYY2	*Pseudomonas sp.*	Oxaloacetate decarboxylase subunit alpha	304.892	186.468	0.01	Transport
61	A0A218M001	*Triticum turgidum*	ATP synthase subunit b, chloroplastic	396.694	390.597	0.04	Transport
62	N1R5T6	*Aegilops tauschii*	ATP synthase delta chain, chloroplastic	275.058	258.572	0.03	Transport
63	G8J3N2	*Pinus discolo*	Ribulose bisphosphate carboxylase large chain	386.564	376.759	0.04	Respiration

Table S4. Relative abundance and functional categorization of down-regulated proteins of wheat plants applied by 40 mg.L^{-1} of TiO2 NPs with *Puccinia striiformis* infection.

No.	Protein IDs	Species	Description	Normalized Average (T3L) mg.L^{-1}	Normalized average (T0L) mg.L^{-1}	P value	Functions
1	M8ASF0	*Triticum urartu*	Thioredoxin	244.786	348.601	0.04	redox
2	A0A1D5U3N1	*Triticum aestivum*	2-dehydro-3-deoxyphosphogluconate aldolase	135.949	156.058	0.02	Aldolase activity
3	M8AYQ8	*Aegilops tauschii*	Basic 7S globulin	84.807	165.469	0.03	Aspartic-type endopeptidase activity
4	A0A1D6BFT5	*Triticum aestivum*	Nickel transport ATP-binding protein NikD	136.140	155.770	0.03	ATP binding
5	B6VCN3	*Triticum monococcum*	Putative vacuolar ATP synthase subunit B isoform 1	234.788	267.904	0.01	ATP binding
6	M7ZV39	*Triticum urartu*	Putative plastid-lipid-associated protein 13, chloroplastic	277.561	341.572	0.01	ATP binding
7	A0A144KR50	*Triticum aestivum*	Ribulose bisphosphate carboxylase activase B	298.567	343.785	0.04	ATP binding
8	Q9SAU8	*Triticum aestivum*	HSP70	369.042	377.451	0.03	ATP binding
9	G3LUU7	*Triticum aestivum*	CytHSP70 (Fragment	384.673	389.562	0.01	ATP binding
10	E7D433	*Triticum aestivum*	EF-hand calcium-binding protein	266.348	300.869	0.02	Calcium binding
11	W5E5Z3	*Triticum aestivum*	Ribulose-phosphate 3-epimerase	153.781	177.563	0.03	Carbohydrate metabolism
12	M8CC18	*Aegilops tauschii*	Phosphoribulokinase	345.009	386.792	0.03	Carbohydrate metabolism
13	A0A1D5UJL4	*Triticum aestivum*	Anaerobic ribonucleoside-triphosphate reductase activating protein	341.673	367.900	0.05	Catalytic activity
14	F9XA11	*Zymos eptoriatritici*	Vacuolar proton pump subunit B	389.341	454.719	0.02	Defense response

15	M8CYL1	*Aegilops tauschii*	Uncharacterized protein	267.794	284.528	0.02	Coenzyme binding
16	Q0WXG8	*Triticum aestivum*	Triticain alpha	278.073	297.893	0.03	Cystein type peptidase activity
17	M8C5I7	*Aegilops tauschii*	Oryzain alpha chain	245.788	277.004	0.03	Cystein type peptidase activity
18	A0A1D5VN83	*Triticum aestivum*	Response regulator receiver protein	267.891	286.702	0.01	DNA Binding
19	A0A1D6S409	*Triticum aestivum*	tRNA-dihydrouridine synthase	175.782	187.987	0.04	FAD binding
20	A0A1D5SGL2	*Triticum aestivum*	Uncharacterized protein	144.890	158.582	0.03	FMN binding
21	Q8LLS8	*Aegilops speltoides*	Phosphoglycerate kinase (Fragment)	256.773	288.893	0.02	Glycolytic process
22	A0A2G1ZD24	*Shewanella sp.*	Fructose-1,6-bisphosphate aldolase	277.579	288.048	0.04	Glycolytic process
23	A0A2G1ZD24	*Shewanella sp.*	Fructose-1,6-bisphosphate aldolase	166.842	178.531	0.02	Glycolytic process
24	A0A1D5VHF9	*Trticum aestivum*	Alpha/beta hydrolase fold protein	189.704	207.983	0.03	Hydrolase activity
25	A0A1D5ZS59	*Triticum aestivum*	Peptidase	347.807	388.561	0.05	Hydrolase activity
26	A0A1D6BGA3	*Triticum aestivum*	TIGR01457 family HAD hydrolase	457.958	466.068	0.04	Hydrolase activity
27	A0A341XUD8	*Tritcum aestivum*	Uncharacterized protein	220.960	265.782	0.03	Not assigned
28	A0A1D5ZB56	*Triticum aestivum*	Uncharacterized protein	345.886	367.009	0.02	Not assigned
29	A0A2A5DPV1	*Candidatus Hydrogenedentes bacterium*	Uncharacterized protein	371.864	389.341	0.04	Not assigned
30	Q9XHL9	*Triticum aestivum*	Histone H1 WH1B.1	388.558	390.676	0.01	Nucleosome assembly
31	N1QZX5	*Aegilops tauschii*	Chlorophyll a-b binding protein, chloroplastic	112.671	156.565	0.04	Photosynthesis

32	FER	*Triticum aestivum*	Ferredoxin, chloroplastic	187.905	188.672	0.02	Photosynthesis
33	M8BMC6	*Aegilops tauschii*	Putative L-ascorbate peroxidase, chloroplastic	276.006	288.675	0.03	Plant defense
34	N1R449	*Aegilops tauschii*	Glycerate dehydrogenase	453.859	488.784	0.03	redox
35	G8J3N2	*Pinus discolo*	Ribulose bisphosphate carboxylase large chain	486.903	506.893	0.01	Respiration
36	RBL	*Triticum aestivum*	Ribulose bisphosphate carboxylase large chain	245.975	277.672	0.01	Respiration
37	A0A1D5ZS57	*Triticum aestivum*	Bifunctional enzyme deaminase/reductase	357.884	377.358	0.01	Riboflavin biosynthetic process
38	A0A1D6S4T3	*Triticum aestivum*	Polyprenyl synthetase	38.953	39.010	0.04	Secondary metabolism
39	W5HK74	*Triticum aestivum*	Vacuolar proton pump subunit B	55.793	66.852	0.02	Secondary metabolism
40	A0A1D5WB89	*Triticum aestivum*	4-diphosphocytidyl-2-C-methyl-D-erythritol kinase	163.892	188.674	0.01	Secondary metabolism
41	A0A1D6BFP3	*Triticum aestivum*	Bifunctional protein FolD	267.923	288.581	0.04	Secondary metabolism
42	H9NAV2	*Triticum aestivum*	Chloroplast Cu/Zn superoxide dismutase	185.842	289.056	0.03	Stress response
43	A0A0D9AXN4	*Pseudomonas fluorescens*	Transposase	177.570	188.049	0.03	Stress response
44	C3VQ52	*Triticum aestivum*	Ascorbate peroxidase (Fragment)	165.002	267.902	0.02	Stress response
45	M8A8P8	*Triticum urartu*	Heat shock cognate 70 kDa protein 4	462.569	476.354	0.01	Stress response
46	Q5QJA6	*Triticum aestivum*	Harpin binding protein	343.856	377.967	0.04	Stress response
47	Q8LRM8|TCTP_	*Triticum aestivum*	Translationally-controlled tumor protein homolog	255.784	277.990	0.04	Stress response
48	M7YF70	*Triticum urartu*	Uncharacterized protein	281.679	364.792	0.01	Stress response
49	A0A1D6A140	*Triticum aestivum*	CalU4	160.083	166.331	0.03	Sulfuric ester hydrolase activity

50	TCTP	*Triticum aestivum*	Translationally-controlled tumor protein homolog	177.486	186.663	0.04	Translation
51	A0A077S298	*Triticum aestivum*	Uncharacterized protein	246.057	277.004	0.02	Translation
52	A0A1D6S9T8	*Tritcum aestivum*	Transporter, auxin efflux carrier (AEC) family protein	266.873	341.691	0.05	Transport
53	M7YQE1	*Triticum urartu*	ADP, ATP carrier protein, mitochondrial	331.672	388.569	0.04	Transport
54	K3W1Z6	*Fusarium pseudograminearum*	Uncharacterized protein	364.601	399.005	0.03	Transport
55	B2W2V5	*Pyreno phora tritici-repentis*	ADP, ATP carrier protein	260.068	289.633	0.05	Transport
56	N1R5T6	*Aegilops tauschii*	ATP synthase delta chain, chloroplastic	178.572	215.950	0.02	Transport
57	A0A1D6CVC4	*Triticum aestivum*	NADH-quinone oxidoreductase subunit N	188.093	197.572	0.02	Transport
58	A0A1D6S9T8	*Triticum aestivum*	Transporter, auxin efflux carrier (AEC) family protein	305.682	331.794	0.01	Transport
